# “Which resilience factors are the most effective for which Outcomes?” A systematic review and Meta-Analysis of multisystemic resilience of children with ADHD

**DOI:** 10.1007/s00787-025-02947-8

**Published:** 2026-01-27

**Authors:** Yves Cho Ho Cheung, Man Ying Kang, Daniel Fu Keung Wong

**Affiliations:** Department of Social Work, Hong Kong Baptist University, Hong Kong, China

**Keywords:** Resilience, Meta-analysis, Systematic review, Attention-deficit/hyperactivity disorder, Child development, Protective factor

## Abstract

Resilience involves multisystemic processes that encompass both personal protective factors and supportive environments, enabling children with attention-deficit/hyperactivity disorder (ADHD) to achieve positive outcomes and thrive beyond expectations. However, ambiguities in the conceptualization and operationalization of resilience have hindered both theoretical and clinical research. This meta-analysis is the first to quantitatively delineate the specific relationship between multisystemic resilience factors and outcomes and propose a conceptual model. A search was conducted in August 2023 and repeated in April 2024 across six databases (PubMed, Scopus, Web of Science, PsycINFO, Embase, and MEDLINE). It identified 28 eligible articles involving 11,622 children aged 18 years or younger. Results indicated that six intrapersonal factors (cognitive functioning, emotional regulation, academic skills, social skills, proactive attitudes and behaviors, and prosocial behaviors) and four interpersonal factors (positive parenting and attachment, parental resources, peer relationships, and other support networks) are associated with improvements in specific outcomes (educational outcomes, wellbeing outcomes, relationship outcomes, internalizing symptoms, and externalizing symptoms) in children with ADHD. This review also revealed that existing literature, which often portrays resilience as lists of factors and focuses primarily on intrapersonal factors, may not adequately capture the dynamic, multisystemic nature and cultural relevance of the resilience process for children with ADHD. Based on our findings, we propose a comprehensive conceptual model to capture four key aspects of resilience: specific outcomes, multisystemic factors, the circularity of factors, and socio-cultural influences. PROSPERO registration number: CRD42023451246.

**Supplementary Information:**

The online version contains supplementary material available at 10.1007/s00787-025-02947-8.

Attention-deficit/hyperactivity disorder (ADHD) affects children worldwide, with a global prevalence rate between 5.9% and 7.1% [1]. Children with ADHD exhibit more antisocial behaviors and peer rejections [2], an increased risk of somatic disorders [1], greater substance use [3], lower academic achievement [4], and reduced quality of life [5]. In the USA, the economic burden on families raising a teenager with ADHD is reported as being five times that of raising a teenager without ADHD [6]. The extra annual societal cost associated with adolescents with ADHD in the USA in 2017–2018 amounted to US$13.8 billion [7].

Although at the population level, ADHD is associated with detrimental childhood outcomes, a closer scrutiny reveals individual differences. “Why do some children appear to adapt better than other children?” Researchers have observed that ordinary human mechanisms, such as cognition, emotion, parenting, and daily social interactions, can guide some, but not all, adversity-exposed children toward developmental trajectories that surpass expectations [8]. Individual differences in the face of adversities have spurred extensive research on resilience.

Over decades of research, the conceptualization of resilience has evolved from viewing it as a personal trait to recognizing it as a combination of bio-psycho-social-ecological factors and processes that conjointly facilitate adaptation [9–11]. This shift represents a significant advancement toward multisystemic models of resilience, examining children’s growth and development amid adversities not only to their personal capabilities but also to the nurturance provided by their families, schools, and the broader socio-ecological context [12].

## Multisystemic resilience factors (RFs) of children with ADHD

Numerous studies have investigated the multisystemic “resilience factors” (RFs) of children with ADHD. At the interpersonal level, research has identified familial and extra-familial RFs linked to either improved favorable outcomes (e.g., school grades, quality of life) or reduced unfavorable outcomes (e.g., conduct problems, depression) in children with ADHD. For example, higher peer acceptance is associated with better school grades and reduced academic impairment [13]; it also buffers the impact of negative parenting on the development of Oppositional Defiant Disorder [14]. Positive student-teacher relationships are linked to enhanced social skills [15]. Better school adjustment predicts lower cigarette smoking rates [16]. Additionally, supportive parenting is negatively related to later emotion dysregulation [17].

At the intrapersonal level, personal RFs of children with ADHD contribute significantly to their improved development. For instance, higher scholastic competence is associated with fewer externalizing and internalizing behaviors and with lower substance use [18]. More prosocial behavior is linked to better student-teacher relationships [19], and participation in extracurricular activities is related to enhanced social relationships [20].

### Current issues in resilience science

Although the findings are promising, several unresolved issues remain in the existing literature on children’s resilience. They include (a) the absence of a conceptual model portraying resilience as processes, (b) the debate over the relative importance of different RFs, and (c) the context-specific nature of resilience.

Despite the identification of more than 60 RFs that foster child development during adversity [21], there is no conceptual model that explains the interactions and dynamic processes among RFs, outcomes, and the contexts in which resilience occurs. Several resilience models [21–24] have been developed within the framework of ecological systems theory [25]. However, these models fail to extend beyond consolidating a list of RFs categorized into different systems (e.g., personal, family, community) and do not provide a comprehensive, concise model that captures the temporal progression and interactions among factors, outcomes, and context.

Another issue revolves around the relative importance of RFs. Research consistently highlights a list of RFs commonly associated with resilience, such as supportive relationships, problem-solving, coping, positive behaviors, and positive cognitions [26]. Nevertheless, the effect sizes of RFs on outcomes reported in existing literature are highly variable [10, 27]. For example, Cicchetti et al. [28] found that personal hardiness has a larger impact when predicting adaptive functioning among children experiencing maltreatment, replacing the protective roles of supportive adults, which are more important for children without a history of maltreatment. Additionally, Dai et al. [29] compared parents rearing children with and without neurodevelopmental disorders (NDDs). They reported that self-kindness was vital in predicting parental stress only among the NDDs group, while coping self-efficacy was crucial in predicting parental stress only among their non-NDDs counterparts.

One reason that may explain the relative importance of RFs for certain outcomes is that their effects are highly context-specific. The existing literature shows that RFs that exhibit a strong effect in one population under specific circumstances may not replicate in another population or situation. For example, Panter-Brick et al. [30] demonstrated this socio-cultural variability in resilience. They found that maternal literacy, which was expected to be an RF, was surprisingly a risk factor in Afghanistan, as it conflicts with local gender norms and is associated with chronic distress in children. Moreover, cultural differences are observed between African and Asian contexts. Social support is the predominant source of resilience of African families rearing children with ADHD [31], but has negligible relevance for Chinese families of children with NDDs [29]. This context-driven phenomenon in resilience science again confirms that a static RF-list approach cannot capture the dynamics and interactions among RF factors, and that a conceptual model of resilience should depict it as a process while also accommodating its socio-cultural heterogeneity.

To the best of our knowledge, there is no empirically developed conceptual model that fulfills the requirements discussed above. This absence applies both to models specifically focused on children with ADHD and to those addressing children in general.

### Research gaps concerning the resilience of children with ADHD

Meanwhile, particularly in relation to resilience in children with ADHD, several research gaps have been observed in the literature. First, despite the identification of numerous RFs, no meta-analysis has been conducted to synthesize existing evidence specifically for children with ADHD quantitatively. Additionally, the latest systematic review on RFs in children with ADHD was published in 2016 [32] and thus requires updating. Conducting such a systematic review and meta-analysis is crucial for empirically developing a list of RFs specifically for children with ADHD, thereby advancing research and interventions that focus on their strengths, resilience, and adaptation.

Second, there is a lack of research delineating which RFs are associated with which outcomes. Various studies have explored different outcomes of children with ADHD, such as school performance, interpersonal relationships, and the reduction of problematic behaviors and emotional disturbances. However, few studies have investigated the specific RF-outcome relationships; such exploration may result in more specific intervention programs or strategies for achieving outcomes specific to children with ADHD.

Third, there is a lack of a conceptual model of resilience processes specifically for children with ADHD. As Ungar [22] emphasizes, resilience researchers need to examine which RFs are best for which outcomes in which contexts. A more comprehensive depiction of resilience should go beyond listing RFs to portray the dynamic processes between RFs and outcomes, elucidating how these processes are influenced by context. Developing this model could help advance the theory and practice in resilience science for children with ADHD.

## The current study

Therefore, this systematic review and meta-analysis aim to achieve three objectives. First, to the best of our knowledge, it is the first meta-analysis to empirically develop a list of existing RFs that contribute to the resilience of children with ADHD. It also updates the systematic review undertaken a decade ago [32]. Second, it delineates the specific relationships and identifies which RFs are significantly associated with which outcomes. Third, it develops a conceptual model of resilience in children with ADHD, as depicted in observational studies, that identifies the processes involved in resilience and may address the socio-cultural influences underlying heterogeneous effect sizes of RFs on outcomes. Such a conceptual model aims to reduce theoretical ambiguities, thereby improving clinical assessments and interventions.

## Method

The study followed the Preferred Reporting Items for Systematic Reviews and Meta-Analyses (PRISMA) guidelines [33] and was registered at PROSPERO (#CRD42023451246).

### Search strategies

Six databases (PubMed, Scopus, Web of Science, PsycINFO, Embase, and MEDLINE) were searched in August 2023 and repeated in April 2024. Our search logic included either the mention of resilience (resilin* OR protect* OR buffer* OR promot* OR cope* OR coping*) or adversity (advers* OR problem* OR difficult* OR issue* OR challeng* OR harm* OR detriment*) to ensure the inclusion of studies identifying any factors potentially associated with better outcomes in the face of adversity. Additionally, search terms included ADHD (ADHD* OR attention-deficit* OR hyperactiv*) and age (child* OR adolescen* OR student* OR teen* OR pediatric* OR youth* OR youngster*).

### Eligibility criteria

A comprehensive bio-psycho-social-ecological model of resilience and its outcomes would involve more than 130 factors [34]. To maintain a more manageable scope in this meta-analysis, we focused on the psycho-social-ecological aspect of resilience by excluding biological RFs (e.g., epigenetics) and biological outcomes (e.g., cardiovascular health). Moreover, non-modifiable demographic factors (e.g., sex and age) were excluded because they are not amenable to intervention and would add complexity to this study without yielding clinical guidance [35]. Additionally, this meta-analysis specifically concentrated on the outcomes for children with ADHD, excluding outcomes related to adults with ADHD or other individuals, such as parents and teachers.

Articles were eligible that met the following criteria: (a) the sample consisted of children under 18 years of age, (b) at least 50% of the children had ADHD, as defined by the Diagnostic and Statistical Manual of Mental Disorders (from DSM-III to DSM-V-TR) [36, 37] or the International Classification of Diseases (from ICD-10 to ICD-11) [38, 39], (c) the measurement of at least one psycho-social-ecological factor that potentially correlated with outcomes of children with ADHD, (d) the measurement of at least one psycho-social-ecological outcome attributed to children with ADHD, and (e) full text written in English. Exclusion criteria were (a) qualitative studies, editorials, reviews, (b) retrospective studies that required adult respondents to report childhood data, and (c) clinical studies that involved treatments or interventions.

After removing duplicate studies, the first and second authors independently screened the articles, with their decisions blinded to each other. Disagreements were resolved through discussion with the third author after full-text screening.

### Data extraction

Data were extracted independently by the first and second authors, and any disagreements were resolved through discussion. Extracted data included: study name, author(s), publication year, publication type, country of sample recruitment, sample size (ADHD, control), percentage of ADHD children in the sample, age range, mean and standard deviation of age, percentage of males, ethnicity, RF variables, and outcome variables. When a variable could be conceptualized as either an outcome or an RF, we followed the classification used by the original authors of the included studies. Correlations (Pearson’s r) between any RF and outcome were extracted as effect size. If a study reported two effect sizes by separating the ADHD and control groups, only data from the ADHD group were extracted. For longitudinal studies, the age at which the RF was measured was extracted and analyzed.

In one study, Pearson’s r was converted from the odds ratio [40, 41]. When conversion was not possible, and effect sizes were not reported, email requests were sent to the study authors. One study reported Spearman’s rho, which was used as the effect size after an unsuccessful attempt to obtain Pearson’s r correlation from the authors. Other studies were excluded if no response was received within two weeks of the request for the additional information or if the authors indicated that the data were unavailable.

### Coding

Outcomes and RFs in the eligible studies were identified during data extraction. The three authors then invited two additional professorial researchers and three doctoral students to further categorize the identified RFs and outcomes through multiple group discussions. These categories served as the units of analysis; in addition, specific RFs were also analyzed that were addressed by at least two eligible studies. For example, the Hierarchical Taxonomy of Psychopathology (HiTOP) model was used as a reference for categorizing internalizing and externalizing symptoms [42]. Studies measuring conduct problems, Oppositional Defiant Disorder, substance abuse, and aggressive behaviors were grouped under the outcome category “externalizing symptoms”, and studies measuring anxiety, depression, and eating disorders were grouped into “internalizing symptoms”. Additionally, the category “wellbeing outcomes”, as defined by Michaelson et al. [43], included assessments of day-to-day emotions, general functioning, life satisfaction, and happiness of children with ADHD. This categorize-and-analyze approach has been used in two prior meta-analysis studies concerning resilience [44, 45]. There was no overlap between instruments used for resilience factors and those used for outcomes; no measure was coded as both. In studies reporting multiple effect sizes within a single category, a mean effect size was calculated using Fisher’s z transformation before data synthesis to address any unit-of-analysis issue [46].

### Quality assessment

The first and second authors independently assessed each eligible study using the Newcastle-Ottawa scale [47, 48]; decisions were blinded to each other. Ratings were assigned on a scale ranging from 0 to 9, with studies categorized as poor quality (scores 0–2), fair quality (scores 3–5), or good quality (scores 6–9). Disagreement was resolved by discussions involving the third author.

### Data synthesis

All statistical analyses were conducted using R [49] with the *meta* and *metafor* packages [50, 51]. Pooled effect sizes were calculated for each pair of *outcome-RF* and each pair of *outcome-RF categories* if at least two eligible studies were identified. Calculations were based on Fisher’s z transformation [52], and then back-transformed to Pearson’s r for reporting purposes. A random effect model was applied for all analyses. Heterogeneity between studies was assessed using I^2^ and Tau^2^ statistics. An I^2^ value of 25%, 50% and 75% indicated low, moderate, and high heterogeneity, respectively [53].

### Sensitivity analyses

To ascertain the robustness of the findings, leave-one-out analyses were applied [54]. Publication bias was assessed by fail-safe N [55] and Egger’s test [56]. Publication bias was negligible if fail-safe N exceeded 5k + 10 or when Egger’s test result was insignificant at a threshold of *α < 0.10*.

## Results

### Eligible studies

**Fig. 1:**
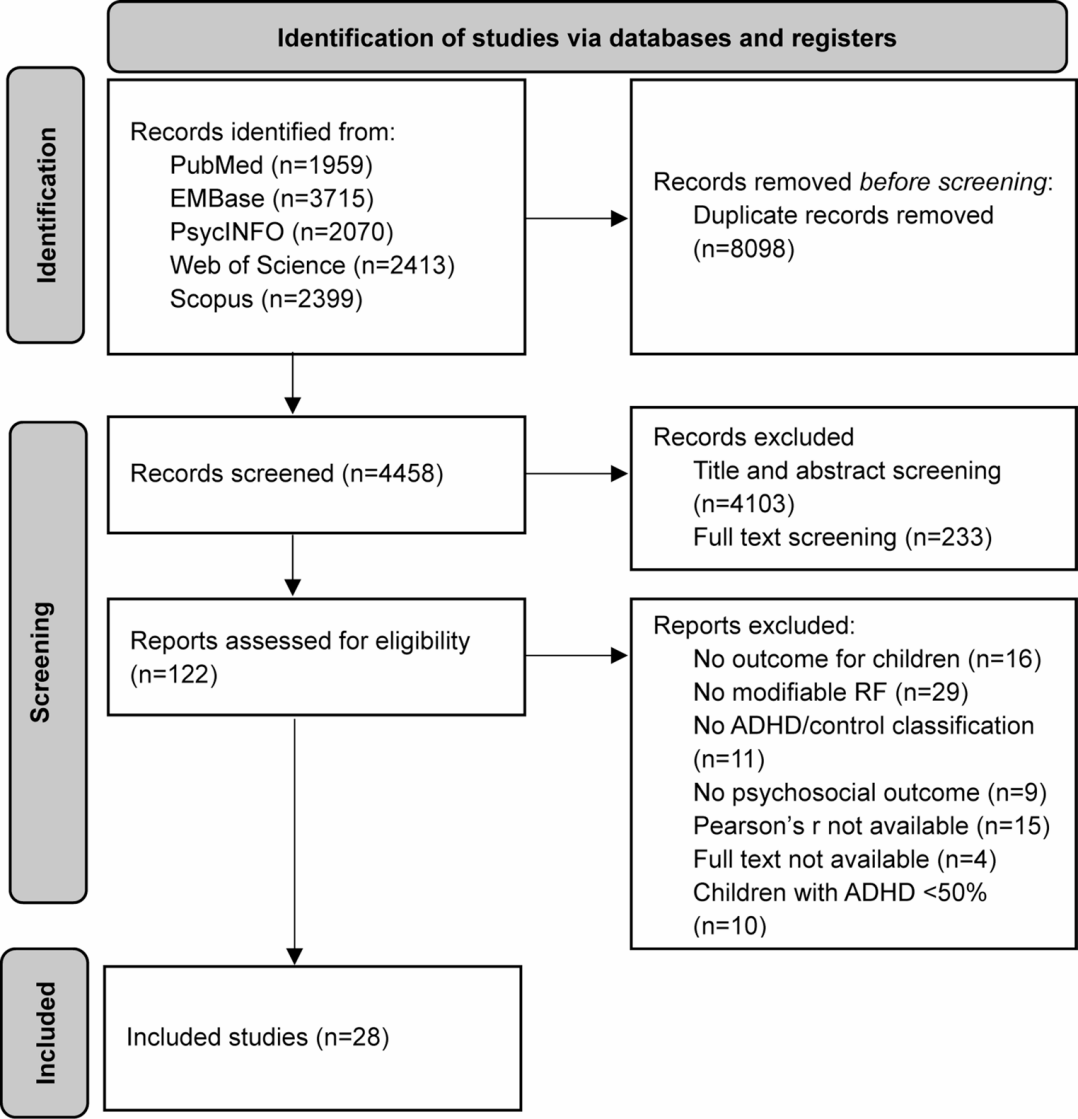
*PRISMA Flow Chart*

The PRISMA flow diagram (Fig. 1) shows the search and screening process. Twenty-eight eligible studies were included after assessing eligibility (Table 1). Exclusion criteria were: a lack of cut-off criteria differentiating children with and without ADHD, less than 50% of their samples comprised children with ADHD, not measuring outcomes for the children but focused on other people, such as parents, not measuring RFs malleable to interventions, not measuring psychosocial outcomes, and unavailable full text or effect sizes.

**Table 1:** *Eligible Studies*

Articles	Country	Sample size			Sample characteristics				Variables investigated	
		ADHD	Control	% ADHD	Age range	Mean Age	% Male	Predominant ethnic group	Outcomes	Resilience factors
Babinski et al. [57]	Canada	120	28	81.1%	(NR)	9.64	77.0%	White	Academic performance	Positive parenting involvement, accepting children’s emotions
Bethune et al. [58]	Canada	209	0	100.0%	5-11	8.15	75.0%	White	Functioning impairment in daily lives	Children’s strengths, parents’ strengths
Cardoos et al. [59]	USA	140	88	61.4%	6-12	(NR)	0.0%	(NR)	Peer victimization	Friendship
Chan et al. [60]	USA	118	70	62.8%	8-13	10.30	67.0%	White	Children’s anxiety	Disciplinary parenting practices, parental confidence
Chronis et al. [61]	USA	108	0	100.0%	4-7	5.25	82.0%	White	Conduct problems	Positive parenting
Duh-Leong et al. [62]	USA	4112	0	100.0%	6-17	10.00	70.0%	White	ADHD, social engagement, and difficulties in friendship	Family cohesion, community support, and social support received by caregivers
Dvorsky et al. [13]	(NR)	93	0	100.0%	10-14	(NR)	72.0%	White	School grade reports, academic impairment	Social skills, social acceptance, and intelligence
Friedman et al. [63]	USA	36	33	52.2%	8-12	9.69	100.0%	White	Math problem solving and computation	Intelligence, short-term memory, and central executive functions
Gaye et al. [64]	USA	120	66	64.5%	(NR)	10.40	66.7%	White	Math performance	Working memory
Granot [65]	Israel	40	25	61.5%	8-14	10.90	49.2%	(NR)	Externalizing symptoms, sociability, internalizing symptoms	Mother-child attachment, teacher-student relationship
Houghton et al. [66]	Australia	42	42	50.0%	10-18	13.40	74.0%	(NR)	Depressive symptoms	Friendship, positive attitude toward loneliness
Jensen et al. [67]	Norway	41	34	54.7%	8-12	9.57	65.3%	(NR)	Emotional lability, ODD symptoms	Working memory
Jia et al. [15]	Canada	213	0	100.0%	6-11	8.58	69.1%	White	Social skills, social problems	Positive parenting, parents’ social competence, teacher-child relationship
Kaypakli et al. [68]	Turkey	150	0	100.0%	12-18	14.45	54.0%	(NR)	ODD symptoms, internet addiction, smartphone addiction, social problems	Stress management
Laslo-Roth et al. [69]	Israel	166	114	59.3%	(NR)	9.66	65.3%	(NR)	Loneliness	Parental hope, parental involvement, family cohesion
Martin [70]	Australia	87	87	50.0%	11-19	(NR)	70.0%	(NR)	School achievement and enjoyment, class participation, intention in schooling involvement	Academic buoyancy
Melnick et al. [71]	USA	48	34	58.5%	(NR)	9.11	100.0%	White	Disruptive behaviors, social acceptance	Emotion regulation, intelligence
Mikami et al. [72]	USA	91	58	61.1%	6-13	9.57	0.0%	White	Depressed/anxious behavior, aggressive behavior	Solitary play, popularity among adults
Mikami et al. [18]	USA	127	82	60.8%	11-18	14.20	0.0%	White	Externalizing behaviors, internalizing behaviors, substance use, eating pathology, and academic achievement	Solitary goal-directed play, popularity among adults, and scholastic competence
Mustafina et al. [73]	Kazakhstan	108	108	50.0%	7-12	9.40	96.0%	(NR)	Peer rejection	Social support received in family and school, dyadic friendship, extracurricular activities, parental warmth, and prosocial behavior
Olczyk et al. [74]	USA	111	92	54.7%	7-12	9.45	61.5%	White	Anxiety	Emotional coping
Rhoads [75]	USA	60	0	100.0%	5-12	9.30	100.0%	White	ODD problems	Interpersonal strengths, intrapersonal strengths, affective strengths, school functioning, positive parenting, family/community involvement, intelligence
Tung et al. [14]	(NR)	81	81	50.0%	5-10	7.30	72.0%	(NR)	ODD problems	Peer acceptance
Ünver et al. [76]	Turkey	60	30	66.7%	(NR)	10.77	63.3%	(NR)	Depressive symptoms, anxiety, and adverse life events	Metacognitive awareness, emotional resilience
Velő et al. [77]	Hungary	79	54	59.4%	6-18	10.24	81.0%	(NR)	Quality of life, peer problems	Prosocial behavior
Volpe et al. [78]	USA	103	43	70.6%	(NR)	8.53	67.8%	White	Academic achievement	Study skills, interpersonal skills, motivation, classroom engagement
Wong et al. [79]	Australia	63	0	100.0%	10-18	14.28	79.3%	White	Quality of life	Coping strategies, treatment coherence
Zhang et al. [80]	USA	3727	0	100.0%	(NR)	12.37	66.0%	White	Childhood flourishment	After school activities, community volunteering, guiding mentor, connected caregiver, safe community, supportive community, resilient family

### Characteristics of eligible studies

Of the 28 eligible studies, one was a doctoral dissertation, and the others were published in peer-reviewed journals. 82% of studies were conducted in Western countries (USA = 13, Australia = 3, Canada = 3, Israel = 2, Hungary = 1, Norway = 1), 11% were conducted in Asia (Turkey = 2, Kazakhstan = 1), and 7% (2 studies) were unspecified. 61% of the studies (*n* = 17) reported the predominant ethnic group of children as White, while ethnicity was unspecified in the remaining studies (39%; *n* = 11). The total sample comprised 11,622 children, with sample sizes in individual studies ranging from 60 to 4,122 *(Mean[SD] = 415.07[992.99])*. The percentage of children with ADHD in the individual study samples ranged from 50% to 100% *(Mean[SD] = 72.49%(20.36%])*. The percentage of males ranged from 0% to 100% *(Mean[SD] = 65.84%[26.48%])*. The mean age of children ranged from 5.25 to 14.45 years *(Mean[SD] = 10.18[2.18])*.

### Quality of studies

The detailed quality assessment results are presented in Online Resource I. Newcastle-Ottawa Scale (NOS) ratings ranged from 3 to 8 across the included studies, with a mean score of 5.25. Based on the established thresholds of NOS ratings [47, 48], 13 studies were classified as fair quality, and 15 as high quality. None was rated as poor quality. Inter-rater reliability showed substantial agreement between two raters (*Cohen’s Kappa = 0.71*).

### Categorization of outcomes

Thirty-three outcomes were identified from the 28 studies, categorized into five categories (Online Resource II). These included externalizing symptoms (*k = 12;* such as conduct problems and aggressive behaviors), internalizing symptoms (*k = 11*; such as depressive and anxiety symptoms), wellbeing outcomes (*k = 10;* such as childhood flourishment and less loneliness), relationship outcomes (*k = 9;* such as social engagement and less peer victimizations), and educational outcomes (*k = 9;* such as school grades and math performance). Externalizing and internalizing symptoms were unfavorable outcomes, whereas wellbeing, relationship, and educational outcomes were favorable ones. 

### Categorization of RFs

Fifty-six RFs were identified in the eligible studies and categorized into 11 categories. They included cognitive functioning (e.g., working memory and executive functioning; *k = 7*), social skills (e.g., social skills and interpersonal strengths; *k = 5*), academic skills (e.g., study skills and scholastic competence; *k = 3*), emotional regulation (e.g., emotional resilience and coping; *k = 5*), proactive attitudes and behaviors (e.g., volunteering and prosocial behavior; *k = 10*), positive parenting and attachment (e.g., parental warmth and mother-child attachment; *k = 7*), disciplinary parenting (e.g., disapproval of child’s emotions and discipline practices; *k = 5*), parental resources (e.g., parental social competence and parental hope; *k = 9*), peer relationship (e.g., friendship and social acceptance; *k = 5*), school support (e.g., student-teacher relationship and school functioning; *k = 5*), and other support networks (e.g., community support and guiding mentor; *k = 5*).

A complete list of the categorization of outcomes and RFs can be found in Tables 1, 2, 3, 4 and 5 in Online Resource II.

### Meta-analysis: effects of RFs on favorable outcomes

**Fig. 2:**
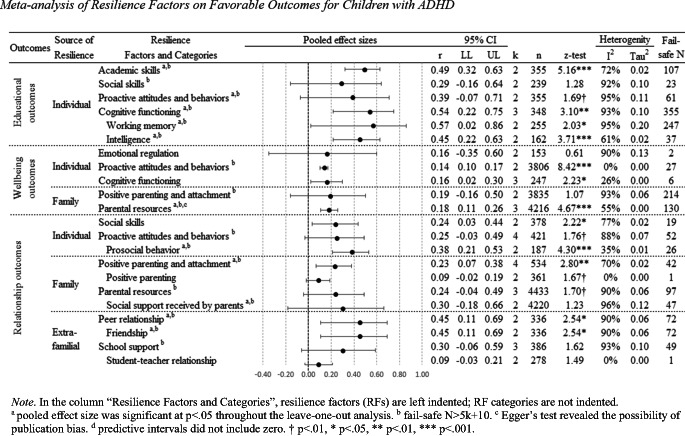
*Meta-analysis of Resilience Factors on Favorable Outcomes for Children with ADHD*

**Fig. 3:**
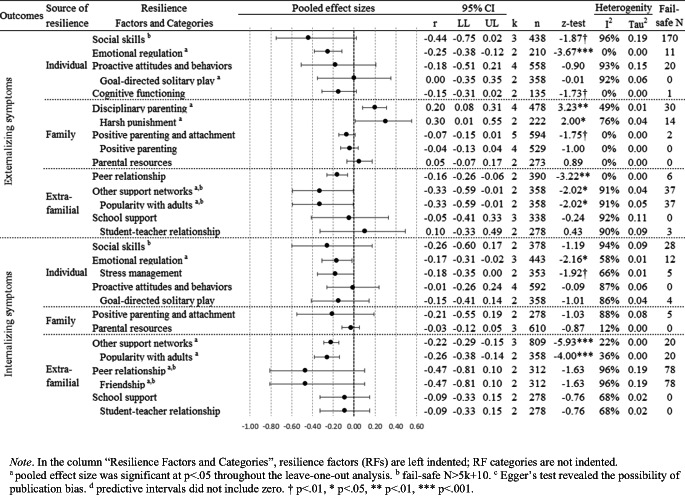
*Meta-analysis of Resilience Factors on Unfavorable Outcomes for Children with ADHD*

All pooled effect sizes, leave-one-out analysis results, fail-safe N, and Egger’s test results depicting how RFs linked to favorable outcomes and unfavorable outcomes are summarized and illustrated in Figs. 2 and 3 (for detailed results, refer to Online Resource III).

The pooled effect sizes on favorable outcomes (Fig. 2) revealed a significant association between three RFs and better educational outcomes. Ranking from the largest correlation to the least, these were “working memory” (r_pooled_ = 0.57, *p* <.05, 95%CI [0.02, 0.86], I^2^ = 95%), “academic skills” (r_pooled_ = 0.49, *p* <.001, 95%CI [0.32, 0.63], I^2^ = 72%), and “intelligence” (r_pooled_ = 0.45, *p* <.001, 95%CI [0.22, 0.63], I^2^ = 61%). The category “cognitive functioning” also showed a significant correlation with educational outcomes (r_pooled_ = 0.54, *p* <.01, 95%CI [0.22, 0.75], I^2^ = 93%).

Wellbeing outcomes were significantly correlated with three categories. Ranked by effect size, these are “parental resources” (r_pooled_ = 0.18, *p* <.001, 95%CI [0.11, 0.26], I^2^ = 55%), “cognitive functioning” (r_pooled_ = 0.16, *p* <.05, 95%CI [0.02, 0.30], I^2^ = 26%), and “proactive attitudes and behaviors” (r_pooled_ = 0.14, *p* <.001, 95%CI [0.10, 0.17], I^2^ = 0%).

Additionally, relationship outcomes were significantly associated with RFs stemming from the children, their families, and their extrafamilial environment. Ranking from the strongest correlations to the weakest, relationship outcomes significantly associated with “friendship” (r_pooled_ = 0.45, *p* <.05, 95%CI [0.11, 0.69], I^2^ = 90%), “prosocial behaviors” (r_pooled_ = 0.38, *p* <.001, 95%CI [0.21, 0.53], I^2^ = 35%), “social skills” (r_pooled_ = 0.24, *p* <.05, 95%CI [0.03, 0.44], I^2^ = 77%), and “positive parenting and attachment” (r_pooled_ = 0.23, *p* <.01, 95%CI [0.07, 0.38], I^2^ = 70%).

### Meta-analysis: effects of RFs on unfavorable outcomes

The pooled effect sizes concerning unfavorable outcomes (Fig. 3) indicate a significant association between lower externalizing symptoms and the RF “popularity with adults” (r_pooled_ = − 0.33, *p* <.05, 95%CI[−0.59, − 0.01], I^2^ = 90%). It also correlated with three categories, “other support networks” (r_pooled_ = − 0.33, *p* <.05, 95%CI[−0.59, − 0.01], I^2^ = 91%), “emotional regulation” (r_pooled_ = − 0.25, *p* <.001, 95%CI[−0.38, − 0.12], I^2^ = 0%), and “peer relationship” (r_pooled_ = − 0.16, *p* <.01, 95%CI[−0.26, − 0.06], I^2^ = 0%).

The RFs associated with internalizing symptoms were similar to those of externalizing symptoms. There was a significant association between lower internalizing symptoms and “popularity with adults” (r_pooled_ = − 0.26, *p* <.001, 95%CI[−0.38, − 0.14], I^2^ = 36%), “other support networks” (r_pooled_ = − 0.22, *p* <.001, 95%CI[−0.29, − 0.15], I^2^ = 22%), and “emotional regulation” (r_pooled_ = − 0.17, *p* <.05, 95%CI[−0.31, − 0.02], I^2^ = 58%). As “popularity with adults” and “emotional regulation” significantly correlated with both internalizing and externalizing symptoms, they may be transdiagnostic factors associated with better mental health.

### Developing a conceptual model of resilience of children with ADHD

**Fig. 4:**
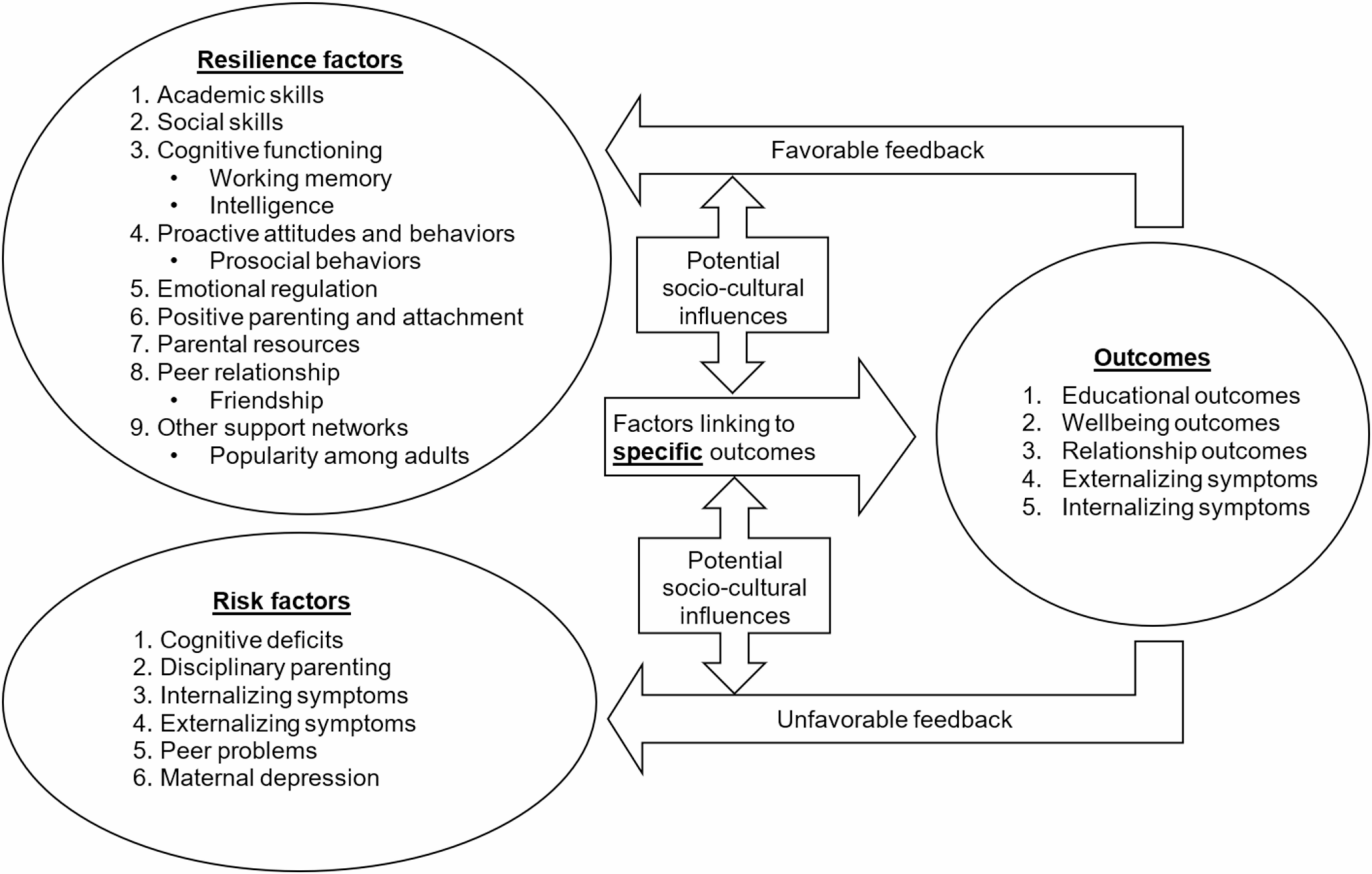
*The Outcome-specific, Multisystemic, Circular, Socio-culturally Influenced Processes of Resilience of Children with ADHD*

Figure 4 presents the conceptual model assembled directly from the meta-analytic findings. The meta-analysis informs the list of resilience factors (upper left in the figure) and the list of outcomes (right). These are “academic skills”, “social skills”, “intelligence”, “working memory” (and the broader category of “cognitive functioning”), “prosocial behaviors” (and the broader category of “proactive attitudes and behaviors”), “positive parenting and attachment”, “parental resources”, and “friendship”, are positively associated with favorable outcomes (i.e., educational, wellbeing, and relationships outcomes). Additionally, “emotional regulation”, “peer relationship”, and “popularity with adults” (and the broader category of “other support networks”) are significantly and negatively associated with unfavorable outcomes (i.e., externalizing symptoms and internal symptoms).

Moreover, our meta-analysis illustrated that each RF is associated with particular outcome(s) rather than all outcomes. Therefore, RFs and outcomes are connected by an arrow labeled “factors linking to **specific** outcomes” in Fig. 4.

## Discussion

This review and meta-analysis synthesized evidence from 28 eligible studies to identify salient resilience factors (RFs) and four characteristics of resilience in children with ADHD. These characteristics are essential to our proposed model of resilience for children with ADHD. This new model has four distinctive features: outcome-specific, multisystemic, circular, and socio-cultural characteristics of resilience. Below is our further deliberation of the model.

### RFs are Outcome-Specific

The meta-analysis empirically identified six personal RFs associated with specific outcomes among children with ADHD: (a) cognitive functioning exhibited a strong positive association with educational outcomes and a small positive association with wellbeing outcomes, (b) emotional regulation was negatively linked to externalizing and internalizing symptoms (with small effect size), (c) academic skills were strongly associated with better educational outcomes, (d) social skills showed a medium positive association with relationship outcomes, (e) proactive attitudes and behaviors showed a small positive association with wellbeing, and (f) prosocial behaviors significantly and positively related to relationship outcomes (medium effect size).

The results underscore that RFs are outcome-specific. For instance, emotion regulation was associated with fewer internalizing and externalizing symptoms, but not with wellbeing, educational, or relationship outcomes. Thus, emotion regulation appears more important for maintaining mental health in children with ADHD than for promoting broader developmental gains. This aligned with the evidence that emotional dysregulation is a core disturbance in ADHD [81]. Likewise, academic skills, social skills, and prosocial behaviors all exhibited significant associations only with their most proximal outcomes. Although cognitive functioning was associated with both educational and wellbeing outcomes, the associations were strong for education and small for wellbeing, indicating that cognitive functioning is primarily an RF for education-related domains. Meanwhile, we observed high heterogeneity in effect sizes for associations between educational outcomes and cognitive functioning (I^2^ = 93%), educational outcomes and working memory (I^2^ = 95%), and relationship outcomes and friendship (I^2^ = 90%). These results may reflect the limited number of studies in some analyses and should be interpreted with caution.

These context-specific relationships between RFs and outcomes highlight the need to develop programs targeting specific RFs with strategies tailored to particular outcomes. Perhaps, a modular approach can be adopted to develop a set of programs that meet the diverse RF needs of children with ADHD.

### Resilience requires multisystemic conceptualization and operationalization

Additionally, the meta-analysis revealed that four interpersonal RF categories contributed significantly to the resilience of children with ADHD: (a) peer relationship exhibited a medium-sized association with better relationship outcomes and a small, negative association with externalizing symptoms, (b) other support networks were associated with lower externalizing and internalizing symptoms, both effects of medium magnitude, (c) positive parenting and attachment were positively related to relationship outcomes (small effect size), and (d) parental resources were positively associated with wellbeing outcomes (small effect size).

It provides empirical evidence that the resilience of children with ADHD is indeed a multisystemic phenomenon involving parents, peers, and other supportive adults, as reflected in the list of RFs in Fig. 4. However, currently, assessment and intervention mainly focus on intrapersonal RF factors and overlook interpersonal RFs. Of the 58 resilience-assessing instruments identified by Terrana et al. [82], at least 21 included no items addressing interpersonal factors. These omissions can lead to “hidden resilience” [83], in which some children who are resilient/vulnerable are mislabeled due to a lack of a multisystemic assessment tool. Indeed, the omission of interpersonal or other systemic levels of RFs deprives children of the opportunity to develop resilience, which can significantly enhance their ability to withstand adversities and strengthen their mental wellbeing. Therefore, future resilience studies should consistently adopt a multisystemic perspective. Assessments and interventions for resilience in children with ADHD should incorporate both intrapersonal, interpersonal, and other systemic RFs to provide a more comprehensive understanding of their personal resources and supportive environments.

### Circular feedback loops suggesting a dynamic process of resilience

Our systematic review found similarities between some outcome variables and RF variables, suggesting that outcomes from previous developmental stages may act as RF factors for the next stage. In our proposed conceptual model (Fig. 4), circularity is represented by favorable and unfavorable feedback, highlighting that outcomes can, in turn, influence both resilience factors and risk factors throughout the developmental process. Therefore, resilience can be conceptualized as ongoing and circular processes involving feedback loops. For instance, social acceptance was an outcome associated with higher emotional regulation of children with ADHD [71]. Meanwhile, an 18-month longitudinal study identified an association between social acceptance, as an RF, and higher school grades and lower academic impairment [13]. It is possible that improving the emotional regulation of children with ADHD may enhance their social acceptance and, in turn, boost their educational outcomes. In the context of child development, it is commonly assumed that any change in a child’s developmental progression will have cascading effects spreading across different aspects of their developmental trajectories. However, while many studies have explored the pathological circular feedback loops leading to children’s mental ill-health, very few have explored the positive and circular feedback loops between RFs and outcomes in resilience [84]. As Bonanno et al. [85] and Ungar et al. [10] have reminded us that resilience is a converging process of numerous RFs on outcomes, it is recommended that future research should explore this circular resilience process, particularly when a quantitative longitudinal research study design with a large sample size is available. On the other hand, qualitative case studies can also be used to investigate the circular resilience processes in a child, complementing the quantitative research to provide a more comprehensive understanding of these dynamics.

### Lack of emphasis on Socio-cultural influences in current resilience research

There is insufficient research with non-Western populations. Most eligible studies (82%) included in our meta-analysis were conducted in Western countries, with White as the predominant ethnicity (61% of eligible studies reporting ethnicity all indicated White was the predominant group, while the remaining 39% failed to specify ethnicity). This suggests a lack of research focused on children with ADHD in non-White populations, such as Asians, Africans, and Latines. Indeed, among the 28 eligible studies reviewed, there is a noticeable lack of research on socio-cultural contexts that may have impacted resilience outcomes. Such an investigation may explain the observed heterogeneity in the significant effects in our meta-analysis (mean I^2^ = 55%), as culture ubiquitously influences how people cope with adversity [26]. For instance, at the interpersonal level, Chinese parents, compared to British parents, exhibit stronger negative emotional reactions to their ADHD children’s hyperactive and impulsive behaviors (but not to inattention) and apply stricter criteria for what they consider non-normative [86]. They tend to discipline their children to minimize problematic behaviors [87]. This culturally influenced parenting style inevitably impacts children’s resilience and mental wellbeing. Another example is that East Asian cultures are generally built on a multi-generational family structure, with caregiving support mainly provided by members within the extended families [88]. In contrast, the African “ubuntu” culture emphasizes human interconnectedness and relies on community support for children with ADHD [37]. Thus, our proposed conceptual model explicitly indicates “potential socio-cultural influences” as moderators that might plausibly modulate the associations between RFs and outcomes as well as the feedback processes.

To conclude, existing literature that emphasizes static models of resilience (i.e., listing RFs) cannot capture the dynamic, multisystemic processes involved in building resilience among children with and without ADHD. A comprehensive conceptual model should include at least the following aspects: specific outcomes, multisystemic RFs, circularity of RFs, and the influences of the socio-cultural context. These are summarized in Fig. 4, including (a) the RFs that showed significant associations with five outcomes in our meta-analysis, (b) the risk factors that we observed in the 28 eligible articles, and (c) the conceptual depiction of the resilience process derived from our study.

### A case illustration of the conceptual model

This conceptual model (Fig. 4) can be illustrated through the hypothetical case of a child with ADHD who has low academic skills but exhibits high emotion regulation ability and social skills. This child’s developmental trajectory encompasses five distinct outcomes: educational attainment, overall wellbeing, relationship quality, and the minimization of both externalizing and internalizing symptoms. Developmental outcomes are continuously influenced by multisystemic risk factors, as observed in the eligible articles, and resilience factors, as indicated by the meta-analysis results.

#### RFs are Outcome-Specific

First, our meta-analysis revealed a key characteristic that these RFs are outcome-specific. For example, emotional regulation has a more pronounced impact on reducing externalizing and internalizing symptoms. Conversely, academic skills are crucial for achieving educational outcomes, and social skills play a vital role in fostering positive relationship outcomes. Consequently, a child with ADHD may exhibit resilience in one outcome while being vulnerable in another. The exemplar child, with their high emotional regulation and social skills, may enjoy good mental health and develop supportive social relationships, despite struggling academically.

#### Circular feedback loops suggesting a dynamic process of resilience

Second, our systematic review further suggests that the resilience process involves circularity. Whether they exceed or fall short of expectations, the five developmental outcomes reciprocally impact RFs, which in turn affect subsequent developmental trajectories. For example, the exemplar child who possesses strong social skills (a personal RF) can develop social relationships with peers and adults (interpersonal outcomes). These social relationships increase their access to peer support and other support networks (interpersonal RFs), thereby lowering their risk of developing internalizing and externalizing symptoms (personal outcomes). This demonstrates that resilience involves circular processes that span both personal and interpersonal elements. Meanwhile, developmental psychopathology research elucidates how feedback of unfavorable outcomes exacerbates risk factors, such as heightening internalizing and externalizing symptoms that may harm the child’s future development [89].

#### Potential Socio-cultural influences

Additionally, our systematic review suggests that the socio-cultural context may influence the effects of RFs on outcomes and the resulting feedback. As discussed above, community support plays a more prominent role in African than in Chinese societies [29, 31]. Consequently, the exemplar child may be better equipped to maintain their mental health in an African context since they have high social skills to solicit support from interpersonal networks. In contrast, Chinese children can strengthen their mental wellbeing through familial support, say, from that provided by their grandparents. Figure 4 includes socio-cultural influences.

### Theoretical implications

Our study has several theoretical and practical implications. The meta-analysis identified specific linkages between RFs and outcomes. It also highlighted the multisystemic nature of these RFs. Additionally, the systematic review suggests conceptualizing resilience as a circular process influenced by socio-cultural context. Based on these findings, we propose a conceptual model of resilience in children with ADHD. The model delineates four key characteristics of their resilience process: (a) specific outcomes, (b) multisystemic factors, (c) the circularity of factors, and (d) socio-cultural influences. This framing moves beyond cataloging factors to capture the temporal, dynamic nature of resilience. Meanwhile, future studies are needed to validate and improve this conceptual model empirically. It would require researching resilience among children from a wider range of ethnic and socio-cultural backgrounds to scrutinize the socio-cultural influences on resilience, and longitudinal data to verify the presence of circularity. Moreover, since we focused on RFs in this study, the proposed risk factors are illustrative and non-exhaustive.

### Practice implications

Regarding practice implications, our meta-analysis empirically revealed the context-specific relationships between RFs and outcomes. These highlight the need to develop programs targeting particular RFs with tailored to specific outcomes. Perhaps, a modular approach can be adopted to develop a set of programs that meet the diverse RF needs of children with ADHD. Each module, with a specific set of objectives, strategies, and skills, will aim to improve specific RFs for the targeted outcomes (e.g., improving academic skills for educational outcomes). In addition, each child with ADHD should be assessed individually to identify their specific training needs and generate a specific package of RF training.

Meanwhile, the importance of socio-cultural influences indicates a need for culturally sensitive practices. Practitioners should consider cultural norms, values, and culture-specific expectations on children and parents when designing and implementing interventions. This is especially vital when working with less-researched non-Western populations to ensure that interventions are relevant and effective for children from diverse backgrounds. Practitioners should adopt a comprehensive assessment approach that evaluates both intrapersonal and interpersonal RFs and other systemic and cultural factors. This holistic view will provide a better assessment of each child’s unique resilience profile and inform more effective intervention planning.

### Study limitations

The study has several limitations. First, the outcomes and RFs measured in the reviewed articles were categorized prior to analysis. For example, variables such as emotion regulation, emotional resilience, coping, stress management, and affective strength were grouped under a single category, “emotional regulation”. Although these variables share some similarities, differences in their assessment across studies could impact the precision of our findings. Moreover, due to a limited number of eligible studies, several findings of our meta-analysis were based on synthesizing two to three articles, and a medium level of heterogeneity was observed across the results. All these issues could lead to a less conclusive but more exploratory result as presented in our meta-analysis. Future meta-analyses should update and verify the findings when more empirical studies on the RFs of children with ADHD are available.

Second, our study included only articles written in English, potentially underrepresenting findings regarding non-English-speaking populations. Furthermore, our study did not include baseline statistics of intervention studies, which may have potentially excluded observational data prior to any manipulation. Future observational studies should be conducted with children from a wider range of ethnic and socio-cultural backgrounds. Future systematic reviews and meta-analyses should aim to broaden their scope by including articles written in languages other than English and baseline data of intervention studies. Moreover, meta-regression analysis should be conducted to elucidate the moderating effects of ethnic and socio-cultural factors when empirical data from children of diverse backgrounds are available.

Third, our study focused solely on the outcomes of children with ADHD, excluding those of other stakeholders such as families and teachers, as well as long-term outcomes in adulthood. Moreover, this study did not consider biological resilience and biological outcomes. Future systematic reviews and meta-analyses should aim to include biological RFs and outcomes to explore the bio-psycho-social-ecological resilience of children with ADHD, as well as including outcomes related to families and teachers, and adulthood outcomes of these children.

Finally, while the mean percentage of children with ADHD in the samples in the reviewed articles was 72.49%, not every respondent had a diagnosis of ADHD. The inclusion of children without ADHD might have influenced our findings. Future observational studies should report statistical findings of children with and without ADHD separately (in the main article or supplementary files). Future systematic reviews and meta-analyses should attempt to differentiate the results of children with and without ADHD.

## Conclusion

To our knowledge, our meta-analysis is among the first to empirically summarize the multisystemic resilience factors (RFs) for children with ADHD and delineate the specific relationships between these factors and outcomes. Additionally, it provides evidence highlighting the significance of interpersonal RFs, supporting the notion that resilience involves multisystemic resources. Meanwhile, our systematic review suggests the presence of circular feedback loops in the resilience process. However, research on non-Western populations and an emphasis on the socio-cultural impact on resilience pathways are inadequate, although existing literature suggests that socio-cultural context could affect the resilience pathways of children with ADHD. We propose a conceptual model delineating four key characteristics of the resilience process of children with ADHD: (a) specific outcomes, (b) multisystemic factors, (c) the circularity of factors, and (d) socio-cultural influences. Future research should adopt longitudinal designs and investigate the cross-cultural differences in resilience among children with ADHD from diverse backgrounds, empirically verifying the presence of circularity and socio-cultural influences.

## Supplementary Information

Below is the link to the electronic supplementary material.

Supplementary Material 1

Supplementary Material 2

Supplementary Material 3

Supplementary Material 4

## Data Availability

No datasets were generated or analysed during the current study.
