# Supplementary material for: “Which resilience factors are the most effective for which Outcomes?” A systematic review and Meta-Analysis of multisystemic resilience of children with ADHD
Source: Eur Child Adolesc Psychiatry. 2026 Jan 27;35(5):1397–413. doi: 10.1007/s00787-025-02947-8 (PMC13272215; doi:10.1007/s00787-025-02947-8)
Supplement: Supplementary file 1 — Supplementary Material 1 [file 787_2025_2947_MOESM1_ESM.docx]

*Quality Assessment*

| Authors | Risk of Bias | | | | |
| --- | --- | --- | --- | --- | --- |
|  | Selection | Comparability | Exposure | Total Score | Risk of Bias |
| Babinski et al. [1] | 2 | 2 | 1 | 5 | Fair |
| Bethune et al. [2] | 2 | 0 | 1 | 3 | Fair |
| Cardoos et al. [3] | 3 | 1 | 2 | 6 | High |
| Chan et al. [4] | 2 | 2 | 2 | 6 | High |
| Chronis et al. [5] | 1 | 2 | 1 | 4 | Fair |
| Duh-Leong et al. [6] | 2 | 2 | 0 | 4 | Fair |
| Dvorsky et al. [7] | 1 | 2 | 1 | 4 | Fair |
| Friedman, et al. [8] | 4 | 2 | 2 | 8 | High |
| Gaye, et al. [9] | 3 | 2 | 1 | 6 | High |
| Granot [10] | 2 | 2 | 0 | 4 | Fair |
| Houghton, et al. [11] | 3 | 2 | 1 | 6 | High |
| Jensen, et al. [12] | 3 | 2 | 1 | 6 | High |
| Jia, et al. [13] | 2 | 2 | 0 | 4 | Fair |
| Kaypakli, et al. [14] | 2 | 0 | 1 | 3 | Fair |
| Laslo-Roth, et al. [15] | 2 | 2 | 2 | 6 | High |
| Martin [16] | 3 | 2 | 1 | 6 | High |
| Melnick, et al. [17] | 3 | 1 | 2 | 6 | High |
| Mikami, et al. [18] | 3 | 1 | 2 | 6 | High |
| Mikami, et al. [19] | 3 | 2 | 2 | 7 | High |
| Mustafina, et al. [20] | 2 | 2 | 1 | 5 | Fair |
| Olczyk, et al. [21] | 3 | 0 | 0 | 3 | Fair |
| Rhoads [22] | 1 | 2 | 1 | 4 | Fair |
| Tung, et al. [23] | 3 | 2 | 3 | 8 | High |
| Ünver, et al. [24] | 3 | 0 | 3 | 6 | High |
| Velő, et al. [25] | 4 | 0 | 2 | 6 | High |
| Volpe, et al. [26] | 3 | 1 | 2 | 6 | High |
| Wong, et al. [27] | 2 | 2 | 1 | 5 | Fair |
| Zhang, et al. [28] | 2 | 2 | 0 | 4 | Fair |

**References**

[1] Babinski DE, Waschbusch DA, King S, Joyce AM, Andrade BF (2017) Maternal and paternal parenting and associations with school performance in a sample of children with varying levels of externalizing behavior problems. Sch Ment Health 9(4):322-333. https://doi.org/10.1007/s12310-017-9229-0

[2] Bethune SC, Rogers MA, Smith D, Whitley J, Hone M, McBrearty N (2023) The impact of internalizing symptoms on impairment for children with ADHD: a strength-based perspective. J Atten Disord 27(1):26-37. https://doi.org/10.1177/10870547221115874

[3] Cardoos SL, Hinshaw SP (2011) Friendship as protection from peer victimization for girls with and without ADHD. J Abnorm Child Psychol 39(7):1035-1045. https://doi.org/10.1007/s10802-011-9517-3

[4] Chan ESM, Macias M, Kofler MJ (2022) Does child anxiety exacerbate or protect against parent–child relationship difficulties in children with elevated ADHD symptoms? J Psychopatholog Behav Assess 44(4):924-936. https://doi.org/10.1007/s10862-021-09922-y

[5] Chronis AM, Lahey BB, Pelham Jr WE, Williams SH, Baumann BL, Kipp H et al (2007) Maternal depression and early positive parenting predict future conduct problems in young children with attention-deficit/hyperactivity disorder. Dev Psychol 43(1):70-82. https://doi.org/10.1037/0012-1649.43.1.70

[6] Duh-Leong C, Fuller A, Brown NM (2020) Associations between family and community protective factors and attention-deficit/hyperactivity disorder outcomes among US children. J Dev Behav Pediatr 41(1):1-8. https://doi.org/10.1097/DBP.0000000000000720

[7] Dvorsky MR, Langberg JM, Evans SW, Becker SP (2018) The protective effects of social factors on the academic functioning of adolescents with ADHD. J Clin Child Adolesc Psychol 47(5):713-726. https://doi.org/10.1080/15374416.2016.1138406

[8] Friedman LM, Rapport MD, Orban SA, Eckrich SJ, Calub CA (2018) Applied problem solving in children with ADHD: the mediating roles of working memory and mathematical calculation. J Abnorm Child Psychol 46(3):491-504. https://doi.org/10.1007/s10802-017-0312-7

[9] Gaye F, Groves NB, Chan ESM, Cole AM, Jaisle EM, Soto EF et al (2024) Working memory and math skills in children with and without ADHD. Neuropsychology 38(1):1-16. https://doi.org/10.1037/neu0000920

[10] Granot D (2016) Socioemotional and behavioural adaptation of students with disabilities: the significance of teacher–student attachment-like relationships. Emot Behav Difficulties 21(4):416-432. https://doi.org/10.1080/13632752.2016.1235324

[11] Houghton S, Lawrence D, Hunter SC, Zadow C, Kyron M, Paterson R et al (2020) Loneliness accounts for the association between diagnosed attention deficit-hyperactivity disorder and symptoms of depression among adolescents. J Psychopathol Behav Ass 42(2):237-247. https://doi.org/10.1007/s10862-020-09791-x

[12] Jensen DA, Høvik MF, Monsen NJN, Eggen TH, Eichele H, Adolfsdottir S et al (2018) Keeping emotions in mind: the influence of working memory capacity on parent-reported symptoms of emotional lability in a sample of children with and without ADHD. Front Psychology 9: 1846. https://doi.org/10.3389/fpsyg.2018.01846

[13] Jia RM, Mikami AY, Normand S (2021) Social resilience in children with ADHD: parent and teacher factors. J Child Fam Stud 30(4):839-854. https://doi.org/10.1007/s10826-021-01907-5

[14] Kaypakli GY, Metin Ö, Varmiş DA, Ray P, Çelik GG, Karci CK et al (2020) Technological addictions in attention deficit hyperactivity disorder: are they associated with emotional intelligence? Indian J Psychiatry 62(6):670-677. https://doi.org/10.4103/psychiatry.IndianJPsychiatry_369_19

[15] Laslo-Roth R, George-Levi S, Rosenstreich E (2021) Protecting children with ADHD against loneliness: familial and individual factors predicting perceived child’s loneliness. Pers Individ Differ 180:110971. https://doi.org/10.1016/j.paid.2021.110971

[16] Martin AJ (2014) Academic buoyancy and academic outcomes: towards a further understanding of students with attention-deficit/hyperactivity disorder (ADHD), students without ADHD, and academic buoyancy itself. Br J Educ Psychol 84(1):86-107. https://doi.org/10.1111/bjep.12007

[17] Melnick SM, Hinshaw SP (2000) Emotion regulation and parenting in AD/HD and comparison boys: linkages with social behaviors and peer preference. J Abnorm Child Psychol 28(1):73-86. https://doi.org/10.1023/A:1005174102794

[18] Mikami AY, Hinshaw SP (2003) Buffers of peer rejection among girls with and without ADHD: the role of popularity with adults and goal-directed solitary play. J Abnorm Child Psychol 31(4):381-397. https://doi.org/10.1023/A:1023839517978

[19] Mikami AY, Hinshaw SP (2006) Resilient adolescent adjustment among girls: buffers of childhood peer rejection and attention-deficit/hyperactivity disorder. J Abnorm Child Psychol 34(6):825-839. https://doi.org/10.1007/s10802-006-9062-7

[20] Mustafina A, Amitov S, Ma JL-C (2022) Multiple levels of factors protecting against peer rejection in children with attention-deficit/hyperactivity disorder. Sage Open 12(1):21582440221082147. https://doi.org/10.1177/21582440221082147

[21] Olczyk AR, Rosen PJ, Alacha HF, Flynn MM (2023) Indirect effect of ADHD on parenting stress through increased child anxiety and decreased emotional regulatory coping. Eur Child Adolesc Psychiatry 33(5):1407-1417. https://doi.org/10.1007/s00787-023-02246-0

[22] Rhoads LKH (2007) The link between attention-deficit/hyperactivity disorder and oppositional defiant disorder: risk and protective factors. Doctor of Philosophy thesis, University of North Carolina.

[23] Tung I, Lee SS (2014) Negative parenting behavior and childhood oppositional defiant disorder: differential moderation by positive and negative peer regard. Aggress Behav 40(1):79-90. https://doi.org/10.1002/ab.21497

[24] Ünver H, Rodopman Arman A, Nur Akpunar Ş (2022) Metacognitive awareness and emotional resilience in children with attention deficit hyperactivity disorder. Scand J Child Adolesc Psychiatr Psychol 10(1):33-39. https://doi.org/10.2478/sjcapp-2022-0003

[25] Velő S, Keresztény Á, Ferenczi-Dallos G, Pump L, Móra K, Balázs J (2021) The association between prosocial behaviour and peer relationships with comorbid externalizing disorders and quality of life in treatment-naïve children and adolescents with attention deficit hyperactivity disorder. Brain Sci 11(4): 475. https://doi.org/10.3390/brainsci11040475

[26] Volpe RJ, DuPaul GJ, DiPerna JC, Jitendra AK, Lutz JG, Tresco K et al (2006) Attention deficit hyperactivity disorder and scholastic achievement: a model of mediation via academic enablers. Sch Psychol Rev 35(1):47-61. https://doi.org/10.1080/02796015.2006.12088001

[27] Wong IY, Hawes DJ, Dar-Nimrod I (2019) Illness representations among adolescents with attention deficit hyperactivity disorder: associations with quality of life, coping, and treatment adherence. Heliyon 5(10): e02705. https://doi.org/10.1016/j.heliyon.2019.e02705

[28] Zhang X, Li Y, Xiao Y, Yu C, Pei Y, Cao F (2024) Association of positive childhood experiences with flourishing among children with ADHD: a population-based study in the United States. Prev Med 179:107824. https://doi.org/10.1016/j.ypmed.2023.107824
