# Supplementary material for: “Which resilience factors are the most effective for which Outcomes?” A systematic review and Meta-Analysis of multisystemic resilience of children with ADHD
Source: Eur Child Adolesc Psychiatry. 2026 Jan 27;35(5):1397–413. doi: 10.1007/s00787-025-02947-8 (PMC13272215; doi:10.1007/s00787-025-02947-8)
Supplement: Supplementary file 2 — Supplementary Material 2 [file 787_2025_2947_MOESM2_ESM.docx]

**Table 1**

*Categorization of Favorable Outcomes Measured in 28 Eligible Articles*

| Categories | Variables included |
| --- | --- |
| Educational  outcomes | School enjoyment  Class participation  Intention to continue education  School grade reports  Language performance  Math performance  Comprehensive academic performance  Academic impairment ^a^ |
|  |  |
| Wellbeing  outcomes | Daily functioning impairment ^a^  Emotional regulation  Emotional dysregulation ^a^  Emotional lability ^a^  Loneliness ^a^  Adverse life events ^a^  Quality of life  Childhood flourishment |
| Relationship  outcomes | Peer problems ^a^  Peer victimization ^a^  Peer rejection ^a^  Social engagement  Sociability  Social capabilities |

*Notes*. ^a^ variables are reverse-coded in data coding, such that positive effect sizes always indicate desirable growth and development

**Table 2**

*Categorization of Unfavorable Outcomes Measured in 28 Eligible Articles*

| Categories | Variables included |
| --- | --- |
| Externalizing  symptoms | Conduct problems  Oppositional defiant disorder  Externalizing behaviors  Substance abuse  Internet addiction  Aggressive behaviors  Disruptive behaviors |
|  |  |
| Internalizing  symptoms | Anxiety symptoms  Depressive symptoms  Internalizing behaviors  Eating disorders |

**Table 3**

*Categorization of Personal Resilience Factors Measured in 28 Eligible Articles*

| Categories | Resilience factors |
| --- | --- |
| Academic skills | Academic skills  Study skills  Academic buoyancy  Scholastic competence |
| Social  Skills | Social skills  Interpersonal strengths |
| Cognitive functioning | Intelligence  Working memory  Short-term memory  Executive functioning  Metacognitive awareness |
|  |  |
| Emotional  regulation | Affective strengths  Coping  Emotional resilience  Emotional regulation  Stress management |
|  |  |
| Proactive attitudes and behaviors | Prosocial behavior  Volunteering  Extracurricular activities involvement  After school activities involvement  Family/community involvement  School motivation  Engagement in classroom  Treatment cohesion  Goal-directed solitary play  Positive attitude toward loneliness |

**Table 4**

*Categorization of Familial Resilience Factors Measured in 28 Eligible Articles*

| Categories | Resilience factors |
| --- | --- |
| Disciplinary parenting | Reduction of child’s emotions  Disapproval of child’s emotions  Rejection of child’s emotions  Negative parenting  Discipline practices |
|  | Harsh punishment |
|  |  |
| Positive parenting and attachment | Support and acceptance of child’s emotion  Positive parenting  Parental warmth  Mother-child attachment |
|  |  |
| Parental resources | Family cohesion  Resilient family  Parental hope  Parental confidence  Parental social competence  Social support received by parents  Parental involvement  Parental strengths  Well-connected caregivers |

**Table 5**

*Categorization of Extrafamilial Resilience Factors Measured in 28 Eligible Articles*

| Category | Resilience factors |
| --- | --- |
| Peer relationship | Social acceptance  Friendship |
|  |  |
| School support | Student-teacher relationship  Social support in schools  Extracurricular activities  School functioning |
|  |  |
| Other support networks | Community support  Guiding mentor  Social support  Safe and supportive community  Popularity with adults |
