# Supplementary material for: “Which resilience factors are the most effective for which Outcomes?” A systematic review and Meta-Analysis of multisystemic resilience of children with ADHD
Source: Eur Child Adolesc Psychiatry. 2026 Jan 27;35(5):1397–413. doi: 10.1007/s00787-025-02947-8 (PMC13272215; doi:10.1007/s00787-025-02947-8)
Supplement: Supplementary file 3 — Supplementary Material 3 [file 787_2025_2947_MOESM3_ESM.docx]

Note:

In the following sections, the pooled effect of each [resilience factor category] on each [outcome category] were shown in RStudio screenshots.

**Effect of [Proactive attitudes & behaviors] on [Educational outcomes]**

**
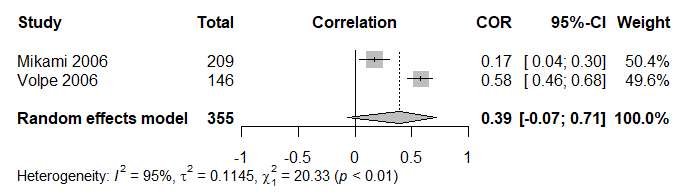
**

**Effect of [Cognitive functioning] on [Educational outcomes]**

**
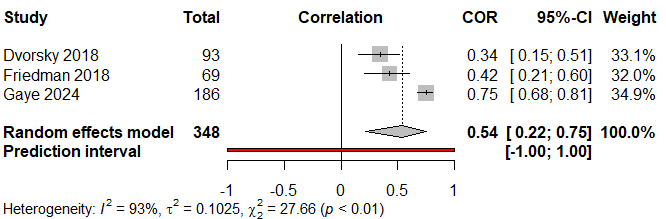
**

Egger’s test result: t = -2.31, df = 1, p-value = 0.2598 Bias estimate: -12.9119 (SE = 5.5817)

**Effect of [Proactive attitudes & behaviors] on [Wellbeing outcomes]**


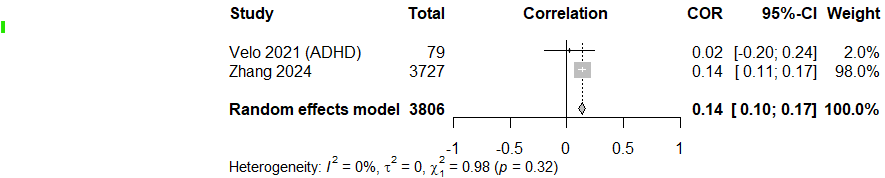


**Effect of [Emotional regulation] on [Wellbeing outcomes]**


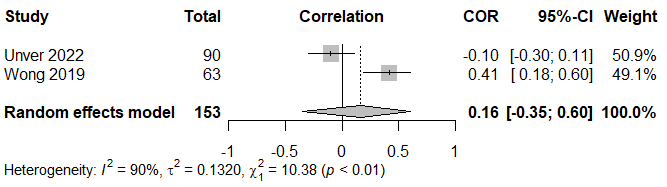


**Effect of [Cognitive functioning] on [Wellbeing outcomes]**


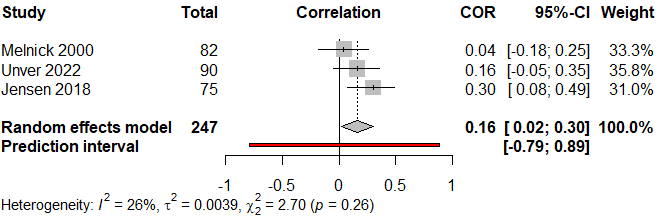


Egger’s test result: t = 0.61, df = 1, p-value = 0.6503 Bias estimate: 12.8168 (SE = 20.9377)

**Effect of [Parental resources] on [Wellbeing outcomes]**


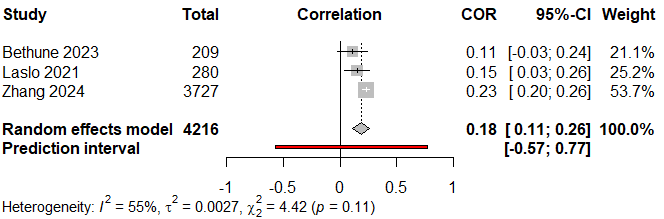


Egger’s test result: t = -9.31, df = 1, p-value = 0.0681 Bias estimate: -2.1028 (SE = 0.2258)

**Effect of [Positive parenting & attachment] on [Wellbeing outcomes]**


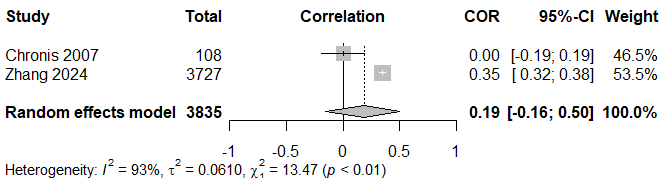


**Effect of [Proactive attitudes & behaviors] on [Relationship outcomes]**


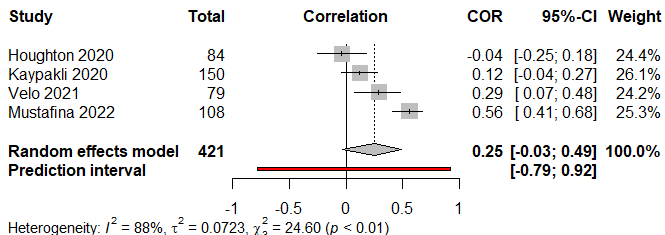


Egger’s test result: t = 0.03, df = 2, p-value = 0.9763 Bias estimate: 0.4421 (SE = 13.1983)

**Effect of [Parental resources] on [Relationship outcomes]**


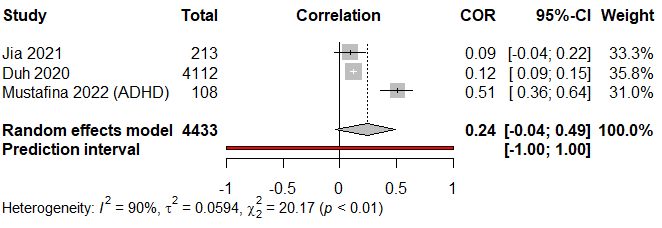


Egger’s test result: t = 0.88, df = 1, p-value = 0.5409 Bias estimate: 2.6892 (SE = 3.0592)

**Effect of [Peer relationship] on [Relationship outcomes]**


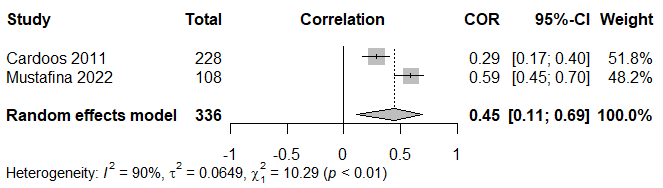


Egger’s test result: t = 4.66, df = 1, p-value = 0.1345 Bias estimate: 10.6210 (SE = 2.2780)

**Effect of [School support] on [Relationship outcomes]**

**
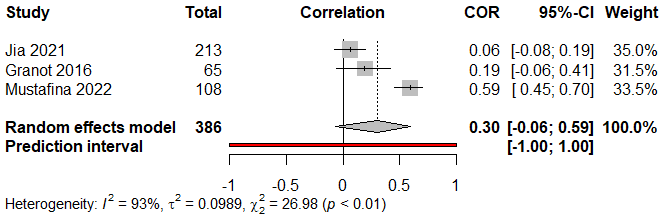
**

Egger’s test result: t = 0.54, df = 3, p-value = 0.6824 Bias estimate: 5.8752 (SE = 10.7850)

**Effect of [Positive parenting & attachment] on [Relationship outcomes]**

**
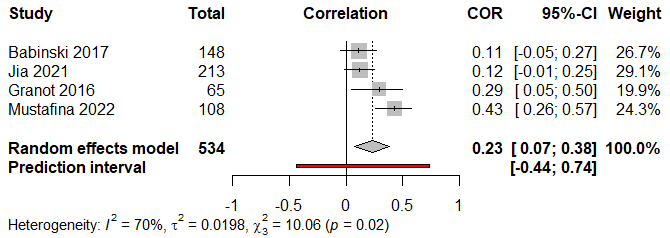
**

Egger’s test result: t = 1.24, df = 2, p-value = 0.3418 Bias estimate: 4.9191 (SE = 3.9789)

**Effect of [Proactive attitudes and behaviors] on [Externalizing symptoms]**

**
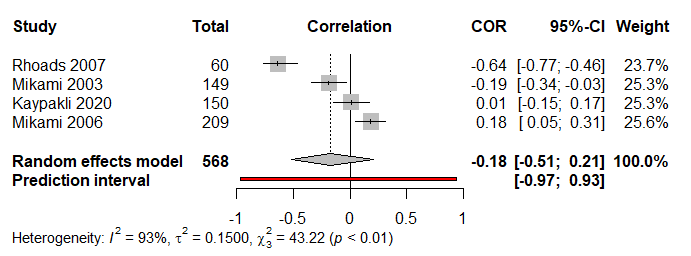
**

Egger’s test result: t = -4.58, df = 2, p-value = 0.0444 Bias estimate: -14.9737 (SE = 3.2665)

**Effect of [Disciplinary parenting] on [Externalizing symptoms]**

**
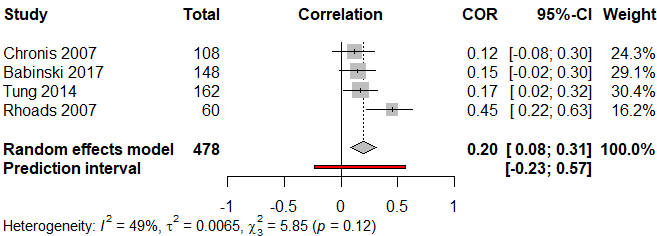
**

Egger’s test result: t = 2.06, df = 2, p-value = 0.1758 Bias estimate: 5.4595 (SE = 2.6521)

**Effect of [Emotional regulation] on [Externalizing symptoms]**

**
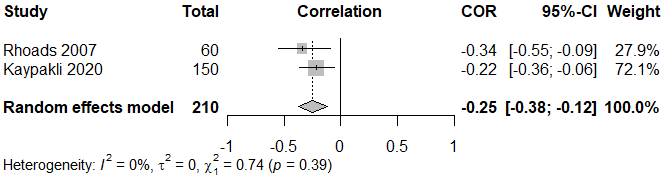
**

**Effect of [Cognitive functioning] on [Externalizing symptoms]**

**
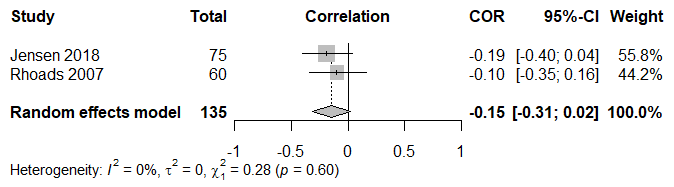
**

**Effect of [Other support network] on [Externalizing symptoms]**

**
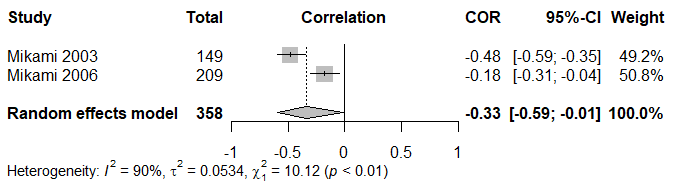
**

**Effect of [Parental resources] on [Externalizing symptoms]**


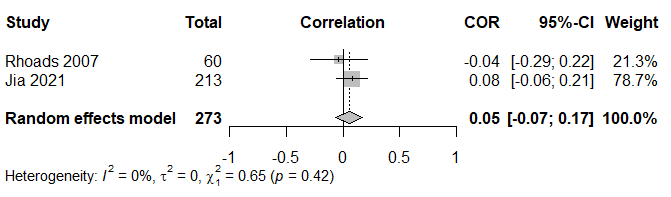


**Effect of [Peer relationship] on [Externalizing symptoms]**


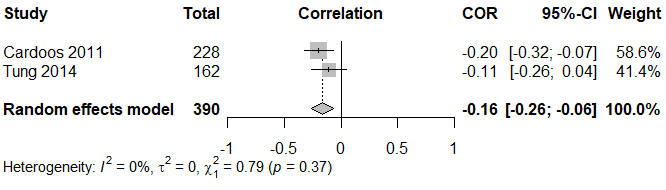


**Effect of [School support] on [Externalizing symptoms]**

**
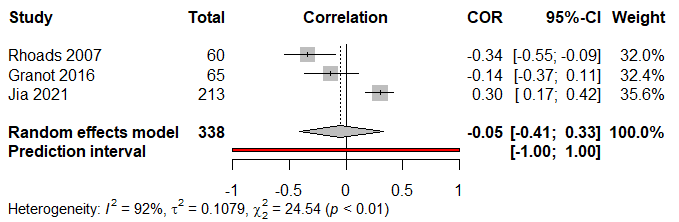
**

Egger’s test result: t = -5.48, df = 1, p-value = 0.1149, Bias estimate: -9.2117 (SE = 1.6810)

**Effect of [Positive parenting & attachment] on [Externalizing symptoms]**


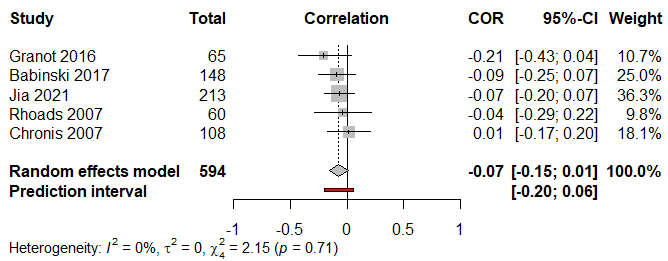


Egger’s test result: t = -0.37, df = 3, p-value = 0.7393 Bias estimate: -0.5590 (SE = 1.5314)

**Effect of [Proactive attitudes & behaviors] on [Internalizing symptoms]**


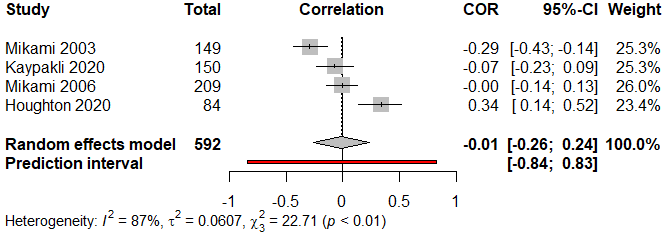


Egger’s test result: t = 0.90, df = 2, p-value = 0.4630 Bias estimate: 8.0905 (SE = 8.9863)

**Effect of [Emotional regulation] on [Internalizing symptoms]**


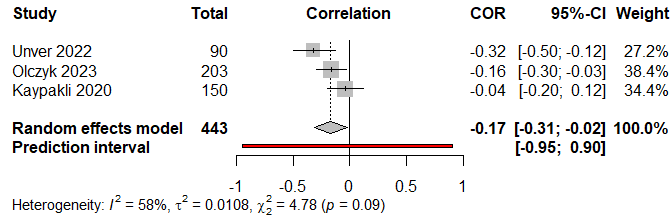


Egger’s test result: t = -0.72, df = 1, p-value = 0.6031 Bias estimate: -4.4914 (SE = 6.2449)

**Effect of [School support] on [Internalizing symptoms]**


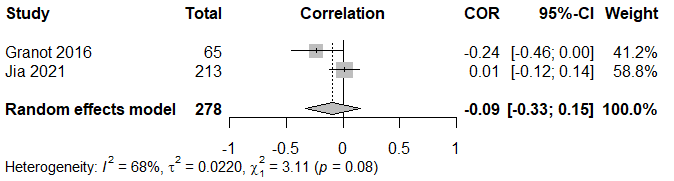


**Effect of [Positive parenting & attachment] on [Internalizing symptoms]**


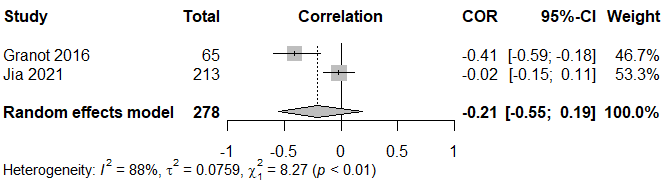


Note:

In the following section, pooled effects of each [resilience factor] on each [outcome] were shown in RStudio screenshots.

**Effect of [Academic skills] on [Educational outcomes]**


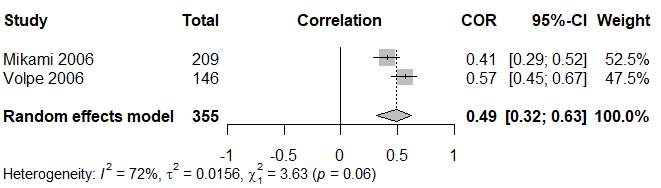


**Effect of [Social skills] on [Educational outcomes]**


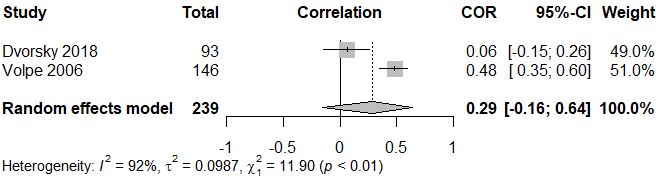


**Effect of [Intelligence] on [Educational outcomes]**


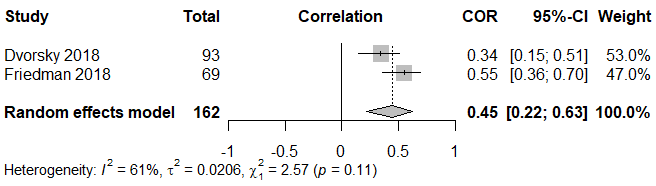


**Effect of [Working memory] on [Educational outcomes]**


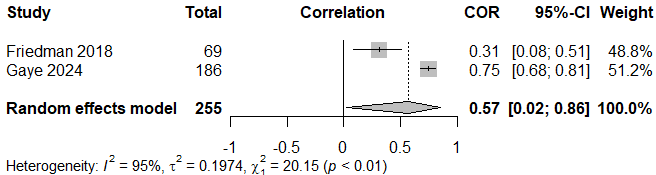


**Effect of [Harsh punishment] on [Externalizing symptoms]**

**
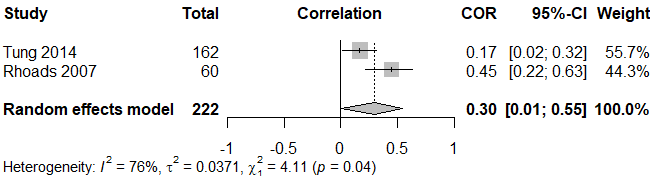
**

**Effect of [Social skills] on [Externalizing symptoms]**

**
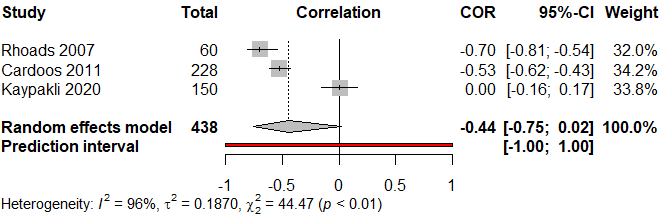
**

Egger’s test result: t = -0.23, df = 1, p-value = 0.8546 Bias estimate: -3.3985 (SE = 14.6173)

**Effect of [Popularity with adults] on [Externalizing symptoms]**

**
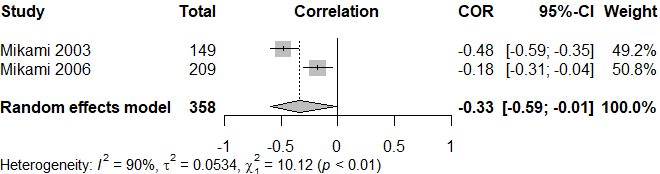
**

**Effect of [Positive parenting] on [Externalizing symptoms]**

**
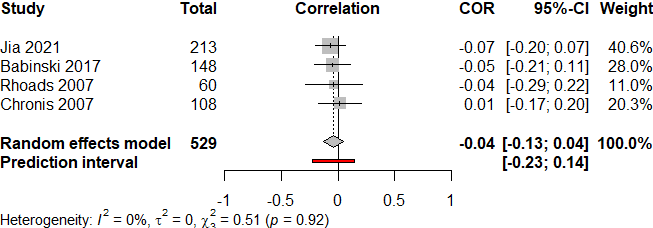
**

Egger’s test result: t = 0.95, df = 2, p-value = 0.4435 Bias estimate: 0.8952 (SE = 0.9451)

**Effect of [Solitary play] on [Externalizing symptoms]**


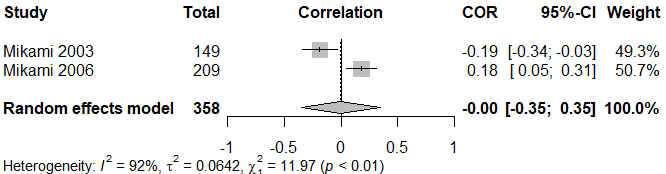


**Effect of [Student-teacher relationship] on [Externalizing symptoms]**

**
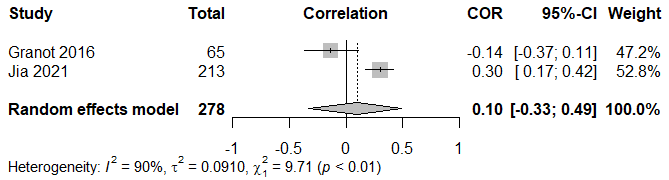
**

**Effect of [Friendship] on [Internalizing symptoms]**

**
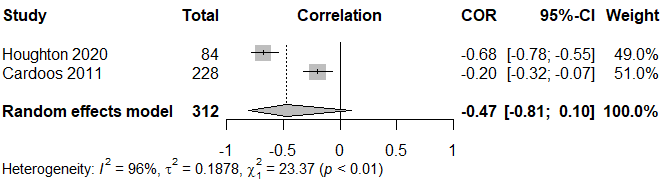
**

**Effect of [Social skills] on [Internalizing symptoms]**

**
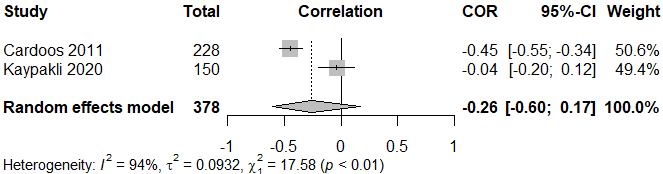
**

**Effect of [Popularity with adults] on [Internalizing symptoms]**

**
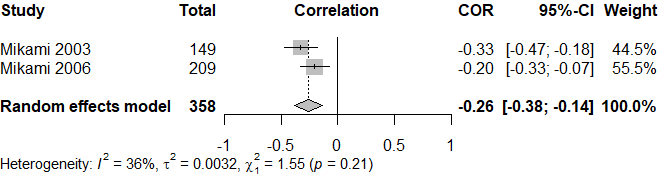
**

**Effect of [Goal-directed solitary play] on [Internalizing symptoms]**

**
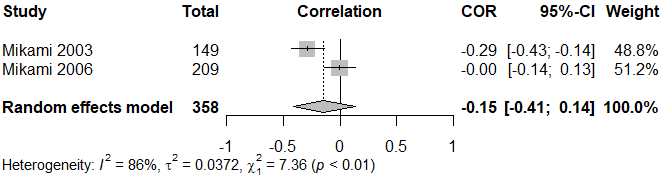
**

**Effect of [Student-teacher relationship] on [Internalizing symptoms]**


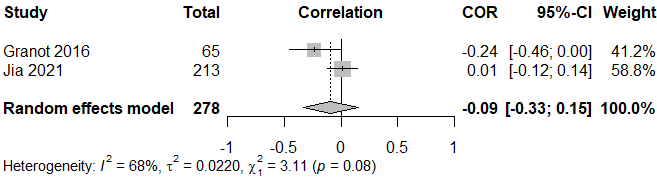


**Effect of [Stress management] on [Internalizing symptoms]**


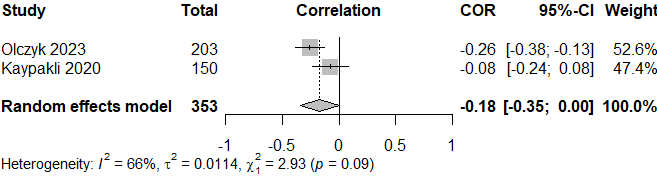


**Effect of [Friendship] on [Relationship outcomes]**


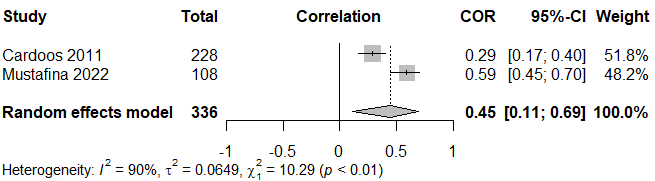


**Effect of [Social skills] on [Relationship outcomes]**


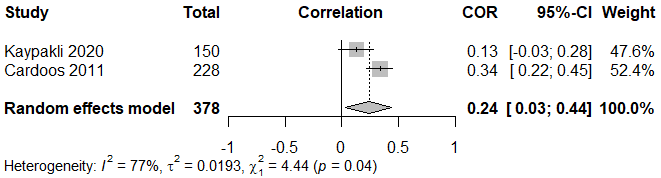


**Effect of [Positive parenting] on [Relationship outcomes]**


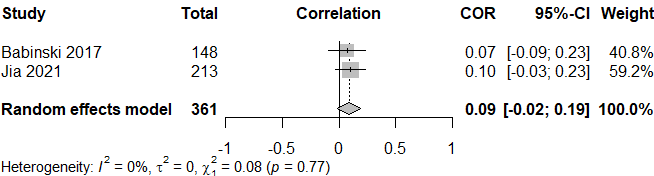


**Effect of [Prosocial behavior] on [Relationship outcomes]**


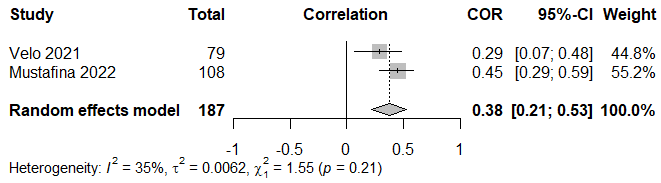


**Effect of [Social support received by parents] on [Relationship outcomes]**

**
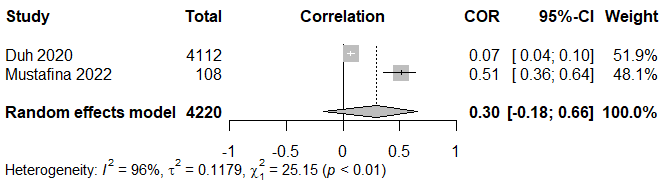
**

**Effect of [Student-teacher relationship] on [Relationship outcomes]**

**
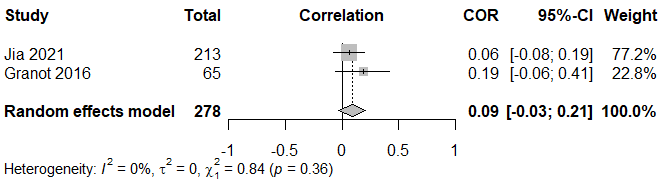
**
