# Supplementary material for: “Which resilience factors are the most effective for which Outcomes?” A systematic review and Meta-Analysis of multisystemic resilience of children with ADHD
Source: Eur Child Adolesc Psychiatry. 2026 Jan 27;35(5):1397–413. doi: 10.1007/s00787-025-02947-8 (PMC13272215; doi:10.1007/s00787-025-02947-8)
Supplement: Supplementary file 4 — Supplementary Material 4 [file 787_2025_2947_MOESM4_ESM.docx]

1. The title was modified to emphasize the development of a conceptual model.
2. The age range of children with ADHD was modiﬁed from 4‐18 years to 0‐18 years. This change was made because most studies in our literature search did not distinguish between young children aged 3 or below and older children aged 4‐18 years.
3. In the protocol, we planned to separate promotive resilience factors (i.e., factors directly associated with better outcomes) from protective resilience factors (i.e., factors that moderate the effects of ADHD on outcomes). However, such separation was not feasible due to the small number of eligible studies and the complexity in analysis and reporting. Therefore, only the direct associations between resilience factors and outcomes were included.
